# Is duration of passive second stage associated with a risk of hysterotomy extension during cesarean?

**DOI:** 10.1371/journal.pone.0258049

**Published:** 2021-10-01

**Authors:** Jade Merrer, Clara Dreyfus, Aude Girault, François Goffinet, Camille Le Ray

**Affiliations:** 1 Clinical Research Unit of Paris Descartes Necker Cochin, APHP, Paris, France; 2 Center of Research in Epidemiology and Statistics/CRESS, Université de Paris, INSERM, (INRA), Paris, France; 3 Department of Obstetrics, Port-Royal Maternity Unit, Cochin Broca Hôtel-Dieu Hospital, Assistance Publique-Hôpitaux de Paris, Paris, France; Lausanne University Hospital: Centre Hospitalier Universitaire Vaudois (CH), SWITZERLAND

## Abstract

**Objective:**

To assess obstetric factors associated with hysterotomy extension among women undergoing a second-stage cesarean.

**Study design:**

This 5-year retrospective cohort study (2013–2017) included all women with second-stage cesarean deliveries of live-born singleton fetuses in cephalic presentation at term. It took place at a tertiary center that practices delayed pushing. We performed univariable and multivariable logistic regression to assess the maternal, obstetric, and neonatal factors associated with hysterotomy extension mentioned in the surgical report. Operative time, postpartum hemorrhage, and maternal complications were also studied.

**Results:**

Of the 3350 intrapartum cesareans, 2637 were performed at term for singleton fetuses in cephalic presentation: 747 (28.3%) during the second stage of labor, 83 (11.1%) of which were complicated by a hysterotomy extension. The median duration of the passive phase of the second stage did not differ between women with and without an extension (164 min versus 160 min, *P* = 0.85). No other second-stage obstetric characteristics, i.e., duration of the active phase, fetal head station, or fetal malposition, were associated with the risk of extension. Factors significantly associated with extension were the surgeon’s experience and forceps use during the cesarean. Women with an extension, compared to women without one, had a longer median operative time (49 min versus 32 min, *P*<0.001) and higher rates of postpartum hemorrhage and blood transfusion (respectively, 30.1% versus 15.1%, *p* = 0.002 and 7.2% versus 2.4%, *P* = 0.03).

**Conclusion:**

The risk of a hysterotomy extension does not appear to be associated with second-stage obstetric characteristics, including the duration of the passive phase of this stage. In our center, which practices delayed pushing, prolonging this passive phase beyond 2 hours does not increase the risk of hysterotomy extension in second-stage cesareans.

## Introduction

Cesarean delivery (CD) is the surgical procedure performed most often worldwide [1] and is associated with several intraoperative and postoperative complications [2–4]. One of these is the occurrence of a hysterotomy extension [2]. This extension can itself be a source of complications during the cesarean underway, by prolonging operative time, increasing blood loss and surgical injuries, but also for subsequent pregnancies, by increasing the risk of uterine rupture [5–10].

The reported frequency of hysterotomy extensions complicating cesareans during labor ranges from 3 to 8% [5, 11, 12]. Compared with cesareans performed during the first stage of labor, those performed during the second stage, that is, at full dilation, are at greater risk of—intended or unintended—extension of the incision [6, 11, 13, 14], with reported rates of 14% to 35% in second-stage cesareans [8, 11–13, 15]. Bligard et al. recently showed that a cesarean during the second stage of labor is the principal risk factor for these extensions [6]. Furthermore, three retrospective cohort studies have assessed the factors associated with its occurrence during second-stage cesareans [8, 12, 15]. Two of them, from the United States, found that a prolonged second stage, that is, exceeding 4 hours, is associated with hysterotomy extension [8, 15]. They did not, however, differentiate between the passive and active phases of the second stage of labor, nor did they take into consideration other obstetric factors related to this stage, such as fetal position or station. Nonetheless, these factors, especially the duration of the passive second stage, may influence the quality of the lower segment and the difficulty in extracting the fetus during the cesarean delivery.

Therefore, we hypothesized that the risk of hysterotomy extension in second-stage cesareans might be associated with the management of this stage of labor, specifically, the duration of the passive second stage before the start of active pushing. Delayed pushing is recommended by the French National Authority for Health to allow fetal descent [16]. Unfortunately, the trials assessing the benefit of delayed pushing have not reported the risk of hysterotomy extension among women with cesareans at full dilation [17–20].

Consequently, our objective was to study the second-stage and intraoperative factors associated with hysterotomy extension, especially the duration of the passive phase of the second stage of labor, among women with cesareans during that stage.

## Materials and methods

We conducted a single-center retrospective cohort study covering the years 2013–2017 at the Port Royal maternity hospital, a public academic tertiary maternity unit in Paris. The National Data Protection Authority (Commission Nationale de l’Informatique et des Libertés, CNIL n° 1755849) approved this study. Under French regulations, the study is exempt from institutional ethics review because it is an observational study using anonymized data from medical records. Women are informed that their records can be used for the evaluation of medical practices and are explicitly informed that they can opt out of these studies.

The study included all women with cesarean deliveries during the second stage of labor of a live-born singleton infant in cephalic presentation at term (≥37 weeks). The cesareans performed during labor were identified from the hospital’s computer database. We analyzed all records, to include only those with a cesarean performed during the second stage of labor. The individual review of all surgical reports enabled the exhaustive collection of the principal endpoint—the occurrence of a hysterotomy extension. All extensions—intended or unintended—that required a suture and were mentioned in the surgical report were counted. Inferior, lateral, and superior extensions were identified separately. Due to the small number of events, they have been grouped together for analysis.

Front-line obstetric care in our unit is managed by midwives. In case of anomalies, such as fetal heart rate abnormalities or failure to progress of dilation, an obstetrician assigned to the delivery unit can intervene. Management of the second stage of labor follows a protocol that includes hourly digital cervical examinations, attempted manual rotation of fetal posterior positions, delayed pushing (starting from 2 hours and up to a maximum of 4 hours after full dilation is reached), and onset of pushing only when the fetal head is engaged. Senior residents under the supervision of a highly trained operator (a fellow or an assistant or a full professor) perform operative vaginal deliveries, using only forceps or spatulas when necessary, for fetuses that have reached the midpelvic station. Cesareans are performed with the Cohen-Stark method, that is, by an anatomical dissection of the planes (subcutaneous tissue, aponeurosis, straight muscles, and parietal peritoneum) by digital spacing without any detachment [21]. The Pfannenstiel and Mouchel techniques can nonetheless be used in women with uterine scars and/or adhesions that prevent the use of the Cohen-Stark technique. The hysterotomy is systematically a transversal segmental incision, extended with fingers (blunt expansion) [22]. Most cesareans are under regional analgesia, and antibiotic prophylaxis is systematically administered. Senior residents, placed to the patient’s left, usually perform this intervention, assisted by a more highly trained operator. When fetal extraction remains difficult, depending on the obstetric situation and the experience of the operator, we attempt to push the fetus back up through the vagina or use vacuum extraction, forceps, or internal podalic version. As a last resort, the operator can deliberately extend the uterine incision, to enable extraction of the fetus.

We studied characteristics related to the management of both labor and the cesarean procedure. Obstetric characteristics associated with management of labor were gestational age, mode of labor onset (spontaneous/cervical ripening/oxytocin induction), oxytocin use, duration of the first stage of labor (from admission to the delivery unit to full cervical dilation), any attempted operative vaginal delivery, attempted manual rotation, fetal station (<0, 0, >0), fetal position at the moment of the cesarean decision (2 categories: anterior or malposition, the latter defined as occiput posterior, deep transverse arrest, or face or brow presentation), duration of the passive second stage, and duration of the active second stage (that is, of pushing). The second stage durations were analyzed first as continuous and then as categorical variables as follows: 4-level variables for the passive second stage (<120/120-179/180-239/≥240 min) and a 3-level variable for the active second stage (<5/5-14/≥15 min). The thresholds for these second-stage durations were defined according to usual practices. We also analyzed the following intraoperative characteristics: experience of the operator supervising the delivery (fellow/assistant professor/full professor), indication for cesarean, pushing of fetus back up through the vagina, and intraoperative maneuvers (Pajot forceps, vacuum extraction, or internal podalic version) for delivery.

Maternal and neonatal characteristics, considered as cofactors, were also recorded: maternal age, pre pregnancy body mass index, geographic origin, parity and number of previous cesarean deliveries, smoking during pregnancy, hypertensive disorders, preexisting or gestational diabetes, birth weight, and head circumference.

Intraoperative outcomes that could be affected by the occurrence of a hysterotomy extension, including operative time, blood loss, sulprostone administration, transfusion, and injuries to nearby organs, were studied as were the postoperative complications included in the medical file (such as postpartum endometritis, abdominal wall infection, and deep abscess), the need for revision surgery during hospitalization, and neonatal outcomes (5-min Apgar score, umbilical cord blood pH, and transfer of the newborn to the neonatal intensive care unit).

A univariable analysis of all the characteristics we studied was performed, with Chi-2 or Fisher’s exact tests, as appropriate, for categorical variables and the Wilcoxon-Mann-Whitney test for continuous variables. Then we performed multivariable logistic regression analysis that included all the variables statistically associated with hysterotomy extension (*P*<0.05) and those that appeared to be clinically relevant for answering the study question, that is, the obstetric factors of the second stage of labor (station and position of the fetal head, and duration of the passive and active phases of the second stage) and birth weight. Because data were missing for less than 3% of each study characteristic, we analyzed the complete cases. The statistical analyses were performed with SAS 9.4 (SAS Institute Inc, Cary, NC, USA).

## Results

**Fig 1:**
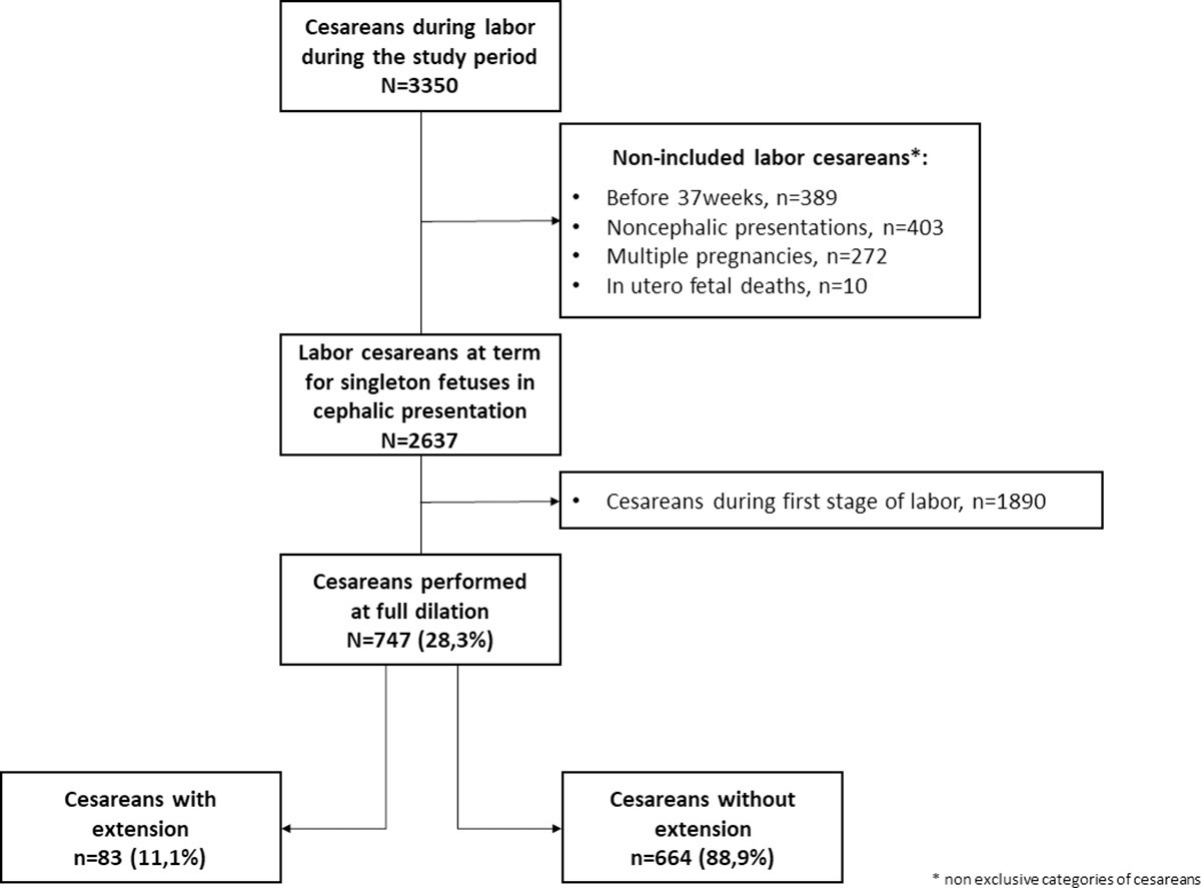
Flow chart.

Of the 3350 cesareans performed during labor during the study period, 2637 were performed at term for singleton fetuses in cephalic presentation. Among these, 747 (28.3%) were performed during the second stage of labor. Hysterotomy extension complicated 83 (11.1%) second stage cesareans (Fig 1). Of these 83 extensions, 34 (41%) involved the left side of the incision and 15 (18%) the right side, while 9 (11%) were bilateral. The extension was vertical cephalad for 12 women (14%), involved the lower segment for 10 (12%), and was cervical for 3 (4%). Only two of these 83 extensions were intentional; all the others occurred unintentionally.

In the univariable analysis, the occurrence of a hysterotomy extension was significantly more frequent among obese women (17.1% vs 14.7% for overweight women vs 9.3 for normal weight women, p = 0.04), those with a hypertensive disease (25.0% vs 10.4%, p = 0.01) (Table 1), and multiparas (respectively 22.3 and 16.2% for multiparas with and without a previous cesarean vs 8.3% for primiparas, p<0.001) (Table 2). The percentage of extensions did not differ according to the duration of the passive second stage: it was 12.3% for durations <120 minutes, 9.6% between 120 and 179 minutes, 10.6% between 180 and 239 minutes, and 11.6% ≥240 minutes (*P* = 0.84) (Table 2). It also did not differ according to fetal head position (15.1 vs 10.2%, *P* = 0.09), or fetal station. None of the other obstetric characteristics we studied was associated with hysterotomy extension. The intraoperative factors associated with extension were the supervisor’s experience and the use of intraoperative maneuvers for delivery. Neither birth weight nor head circumference was associated with hysterotomy extension (Table 3).

**Table 1:** Frequency of incision extensions according to maternal characteristics.

		Extension	No extension	p-value*
		n = 83	n = 664	
		n (%)	n (%)	
**Maternal age (years)**				0.20
	< 25	5 (14.7)	29 (85.3)	
	25–34	42 (9.8)	388 (90.2)	
	35–40	26 (15.1)	146 (84.9)	
	< 40	10 (9.0)	101 (91.0)	
**Body Mass Index (Kg/m^2^)** ^**a**^				0.04
	< 25	49 (9.3)	476 (90.7)	
	25–29	20 (14.7)	116 (85.3)	
	≥ 30	14 (17.1)	68 (82.9)	
**Geographic origin** ^**b**^				0.89
	Metropolitan France	41 (11.1)	329 (88.9)	
	French overseas departments	2 (10.5)	17 (89.5)	
	Europe	5 (8.3)	55 (91.7)	
	North Africa	14 (13.1)	93 (86.9)	
	Sub-saharan Africa	7 (9.6)	66 (90.4)	
	Asia	8 (15.7)	43 (84.3)	
	Other	2 (7.7)	24 (92.3)	
**Smoking** ^**c**^				0.31
	Yes	10 (14.5)	59 (85.5)	
	No	70 (10.5)	599 (89.5)	
**Hypertensive disorders**				0.01
	Yes	9 (25.0)	27 (75.0)	
	No	74 (10.4)	637 (89.6)	
**Diabetes**				0.83
	Yes	13 (11.7)	98 (88.3)	
	No	70 (11.0)	566 (89.0)	

*p-value by Chi square or Fisher’s exact test as appropriate.

Results are presented in rows.

Missing values

^a^n = 4

^b^n = 41

^c^n = 9.

**Table 2:** Frequency of incision extensions according to obstetrical characteristics.

		Extension	No extension	p-value*
		n = 83	n = 664	
		n (%)	n (%)	
**Parity and previous cesarean**				<0.001
	Primiparas	47 (8.3)	517 (91.7)	
	Multiparas without previous cesarean	13 (16.2)	67 (83.8)	
	Multiparas with previous cesarean	23 (22.3)	80 (77.7)	
**Gestational age (weeks)** ^**a**^		40.3 (39.4–41.0)	40.1 (39.1–41.0)	0.62
**Mode of labor onset**				0.71
	spontaneous	50 (10.4)	430 (89.6)	
	cervical ripening	25 (12.5)	175 (87.5)	
	induction	8 (11.9)	59 (88.1)	
**Oxytocin during labor**				0.97
	Yes	72 (11.1)	575 (88.9)	
	No	11 (11.0)	89 (89.0)	
**Duration of the first stage (min)** ^**a**^		390 (285–560)	450 (270–585)	0.52
**Duration of the passive second stage (min)** ^**a**^ ^,^ ^**b**^		164 (73–210)	160 (67–212)	0.85
	<120	33 (12.3)	235 (87.7)	0.84
	120–179	14 (9.6)	132 (90.4)	
	180–239	30 (10.6)	254 (89.4)	
	≥ 240	5 (11.6)	38 (88.4)	
**Duration of active second stage (min)** ^**a**^ ^,^ ^**c**^		5 (0–10)	5 (2–10)	0.11
	< 5	26 (12.7)	178 (87.3)	0.56
	5−14	42 (10.9)	343 (89.1)	
	≥ 15	14 (9.2)	139 (90.8)	
**Attempted operative vaginal delivery** ^**d**^				0.10
	Yes	0 (0.0)	23 (100.0)	
	No	82 (11.4)	637 (88.6)	
**Attempted manual rotation** ^**e**^				0.08
	No	46 (10.0)	414 (90.0)	
	Failure	21 (17.1)	102 (82.9)	
	Success	16 (10.1)	142 (89.9)	
**Fetal station**				0.48
	< 0	79 (11.5)	605 (88.5)	
	0	4 (8.0)	46 (92.0)	
	< 0	0 (0.0)	13 (100.0)	
**Fetal head position** ^**f**^				0.09
	anterior	60 (10.2)	528 (89.8)	
	occiput posterior or other^g^	23 (15.1)	129 (84.9)	

*p-value by Chi square or Fisher’s exact test as appropriate.

Results are presented in rows.

^a^ Values are given as median (interquartile range).

Missing values

^b^n = 6

^c^n = 5

^d^n = 4

^e^n = 6

^f^n = 7.

^g^ Deep transverse arrest, face or brow presentation.

**Table 3:** Frequency of hysterotomy extensions according to intraoperative and neonatal characteristics.

		Extension	No extension	p-value*
		n = 83	n = 664	
		n (%)	n (%)	
**Supervisor’s experience** ^**a**^				0.06
	Fellow	64 (12.7)	441 (87.3)	
	Assistant professor	9 (5.8)	147 (94.2)	
	Full professor	9 (11.0)	73 (89.0)	
**Indication for cesarean** ^**b**^				0.67
	Failure to progress	41 (11.5)	316 (88.5)	
	FHR abnormalities	42 (11.1)	336 (88.9)	
	Other	0 (0.0)	12 (100.0)	
**Pushed back through the vagina**				0.08
	Yes	7 (21.2)	26 (78.8)	
	No	76 (10.6)	638 (89.4)	
**Intraoperative maneuver**				0.03
	No	74 (10.7)	616 (89.3)	
	Vacuum extraction	2 (7.1)	26 (92.9)	
	Forceps	3 (50.0)	3 (50.0)	
	Internal podalic version	4 (17.4)	19 (82.6)	
**Birthweight (g)**				0.67
	< 3500	44 (11.4)	343 (88.6)	
	3500–3999	32 (11.6)	243 (88.4)	
	≥ 4000	7 (8.2)	78 (91.8)	
**Head circumference (cm)**				0.89
	< 34	10 (11.8)	75 (88.2)	
	34–36	56 (10.7)	468 (89.3)	
	≥ 36	16 (12.0)	117 (88.0)	

*p-value by Chi square or Fisher’s exact test as appropriate.

Results are presented in rows.

^a^ Missing values: n = 4.

^b^ Other indications of cesarean deliveries: brow presentations (n = 8), face presentation (n = 2), cephalopelvic disproportion (n = 1), herpetic recurrence diagnosed during the second stage (n = 1).

Abbreviation: FHR, Fetal Heart Rate.

In the multivariable analysis, no characteristic of the second stage of labor, including the duration of its passive phase, was associated with hysterotomy extension (Table 4). For the intraoperative variables, the odds of hysterotomy extension were higher with forceps (adjusted odds ratio [aOR], 15.5, 95% CI 2.2, 107.4) and when a fellow, versus an assistant professor, supervised the surgery (aOR, 2.9 95% CI, 1.4, 6.3).

**Table 4:** Risk factors for incision extension during the second stage of labor and cesarean delivery—crude and adjusted odd ratios.

		Hysterotomy extension	
		Crude OR (95% CI)	Adjusted OR (95% CI)^a^
**Duration of the passive second stage (min)**			
	<120	Ref –	Ref –
	120–179	0.8 (0.4–1.5)	0.7 (0.4–1.5)
	180–239	0.8 (0.5–1.4)	1.3 (0.7–2.4)
	≥ 240	0.9 (0.3–2.6)	1.3 (0.5–4.1)
**Duration of active second stage (min)**			
	< 5	1.2 (0.7–2.0)	1.1 (0.6–1.9)
	5−14	Ref –	Ref –
	≥ 15	0.8 (0.4–1.6)	1.0 (0.5–1.9)
**Fetal occiput posterior or other position** ^**b**^		1.7 (0.9–2.6)	1.5 (0.8–2.8)
**Fetal station ≥ 0**		0.5 (0.2–1.5)	0.6 (0.2–1.8)
**Intraoperative maneuver**			
	No	Ref –	Ref –
	Vacuum extraction	0.6 (0.1–2.8)	0.7 (0.2–3.1)
	Forceps	**8.3 (1.7–42.0)**	**15.5 (2.2–107.4)**
	Internal podalic version	1.8 (0.6–5.3)	1.1 (0.3–4.3)
**Supervisor’s experience**			
	Fellow	**2.4 (1.2–4.9)**	**2.9 (1.4–6.3)**
	Assistant professor	Ref –	Ref –
	Full professor	2.0 (0.8–5.3)	2.2 (0.8–6.1)

^a^ Adjusted for body mass index, parity and previous cesarean, hypertensive disease during pregnancy and birthweight.

^b^ Deep transverse arrest, face or brow presentation.

Abbreviation: OR, odds ratio; CI, confidence interval.

In addition, operative time was longer (49 min [IQR 40, 60] vs 32 min [IQR 28, 40] *P*<0.001) and the rates of postpartum hemorrhages (30.1% vs 15.1%, *P* = 0.002), transfusions (7.2% vs 2.4%, *P* = 0.03) and injuries to neighboring organs (19.3% vs 1.6%, *P*<0.001) were all higher among the women with hysterotomy extensions (Table 5). The existence of an extension was not significantly associated with poor neonatal outcomes.

**Table 5:** Maternal and neonatal complications according to the occurrence of hysterotomy extension.

		Extension	No extension	p-value*
		n = 83	n = 664	
		n (%)	n (%)	
**Duration of surgery (min)** ^**a**^ ^,^ ^**b**^		49 (40–60)	32 (28–40)	<0.001
	< 29	5 (6.0)	173 (26.5)	<0.001
	29–33	4 (4.8)	178 (27.3)	
	34–40.4	16 (19.3)	176 (26.9)	
	≥ 40.5	58 (69.9)	126 (19.3)	
**Estimated blood loss (mL)** ^**c**^				0.002
	< 500	58 (69.9)	562 (84.9)	
	500–999	21 (25.3)	90 (13.6)	
	≥ 1000	4 (4.8)	10 (1.5)	
**Sulprostone**		9 (10.8)	3 (6.5)	0.14
**Transfusion**		6 (7.2)	16 (2.4)	0.03
**Injuries to nearby organs**				<0.001
	No	67 (80.7)	653 (98.4)	
	Bladder	2 (2.4)	6 (0.9)	
	Arterial	14 (16.9)	5 (0.7)	
**Infectious complications**				0.15
	No	78 (94.0)	647 (97.4)	
	Parietal	3 (3.6)	10 (1.5)	
	Endometritis	2 (2.4)	5 (0.8)	
	Other^d^	0 (0.0)	2 (0.3)	
**Surgical revision** ^**e**^		3 (3.6)	12 (1.8)	0.23
**5-min Apgar score < 7**		7 (8.4)	33 (5.0)	0.19
**Umbilical cord blood pH**				0.29
	< 7.00	5 (6.0)	21 (3.2)	
	7.00–7.09	9 (10.8)	60 (9.2)	
	≥ 7.10	69 (83.2)	574 (87.6)	
**Neonatal transfer**				0.94
	No	78 (94.0)	616 (92.8)	
	Mother-child unit	2 (2.4)	16 (2.4)	
	Intensive care unit	3 (3.6)	32 (4.8)	

*p-value by Chi square or Fisher’s exact test as appropriate.

Results are presented in columns.

^a^ Values are given as median (interquartile range).

Missing values

^b^n = 11

^c^n = 2 disproportion (n = 1), herpetic recurrence diagnosed during the second stage (n = 1).

^d^Other infectious complications: sepsis of undetermined etiology (n = 1), pleuro-pneumonitis (n = 1).

e Indications for surgical revision: wall abcess (n = 5), wall hematomas (n = 3), hematoma of the broad ligament with hemoperitoneum (n = 1), uterine revision with placement of Bakri balloon (n = 1), deep abcess (n = 1), eventration (n = 1) and no indication (n = 3).

## Discussion

In this five-year retrospective cohort study, we did not observe an association between the duration of the passive second stage of labor and the risk of hysterotomy extension. No obstetric characteristic of the second stage was associated with a higher risk of extension.

Our study presents several strengths. First, we analyzed a large cohort of women with cesareans during the second stage of labor. We collected data from the medical records that enabled us to examine many second-stage and intraoperative factors potentially associated with the risk of a hysterotomy extension, in particular, the duration of the passive second stage. Moreover, all surgical reports were examined twice to ensure accurate identification of our primary endpoint.

Our study also had some limitations, particularly due to the retrospective design. We cannot rule out residual confounding related to factors that are not routinely collected in surgical reports. For example, the reports do not specify if the blunt (finger) incisions were performed in a cephalad-caudad direction (myometrial gap between high and low) (cephalad-caudad expansion) or transversally (transversal gap of the myometrium) (transversal expansion). The literature has shown that cephalad-caudad expansions are at lower risk of hysterotomy extension than transversal expansions [23]. Another study published in 2016 seems to point in this direction, but without showing any significant difference [24].

We found that 11% of the cesarean deliveries performed during the second stage of labor were complicated by hysterotomy extensions. This rate was lower than those reported in the literature [8, 11–13, 15]. We cannot rule out the possibility that the surgical reports understated the number of extensions in our study; those included might represent only the most severe extensions, those requiring complicated surgical repair. The most severe extensions are those potentially associated with more hemorrhagic complications. Our lower rate of extensions could also be explained by different types of obstetric management between the studies. For example, in our hospital, if the fetal head is not engaged, we do not usually initiate pushing. Accordingly, our cesarean rate during the second stage after instrument failure is low (3%), which may partially explain our lower rate of extensions. This also explains our high rate (28.3%) of cesareans during the second stage of labor as well as our failure to find a link between fetal station and hysterotomy extension (only 15 women had a station > 0). In a French series reporting 412 cesareans at full dilation including 81 (19.6%) performed after failed instrumental delivery, the extension rate was 22% [14].

In our study, the duration of the passive second stage was not associated with the occurrence of hysterotomy extension, even for passive second-stage durations exceeding 3 and even 4 hours. This result is reassuring regarding this practice, which in some cases allows fetal engagement and vaginal delivery [18, 19]. Two studies from the USA found more hysterotomy extensions when the second stage exceeded 4 hours (respectively 40 vs 26% in Sung et al. and 33 vs 17% in Isquick et al.) but did not specify whether pushing was early or delayed. It is therefore impossible to know whether the passive phase of the second stage, the active phase, or both were associated with a higher risk of extension [8, 15]. We can assume, however, that in these American studies, early pushing was the rule. It is therefore difficult to compare their results with ours.

Three meta-analyses have shown that the risk of hysterotomy extension decreases when internal podalic version with breech extraction is performed [25–27]. In our study, the use of forceps during cesarean delivery was the only intraoperative maneuver significantly associated with hysterotomy extension. Neither vacuum extraction nor internal podalic version was associated with extension. But the reason for the choice of one technique rather than another was not specified in the surgical reports. We therefore cannot rule out an indication bias: it is possible that forceps were used in situations where the operative intervention was the most difficult, especially with a deeply impacted fetal head. Our results do not allow us to conclude that there are benefits to vacuum extraction when operative intervention is difficult; this is in line with the results from a recent meta-analysis that did not report any difference in the use of forceps or vacuum extraction in the occurrence of hysterotomy extension [27].

Finally, fetal station at the moment of the cesarean delivery was not associated with the occurrence of extensions. This result does not support the use of devices such as the Fetal Pillow (Safe Obstetric Systems, Brentwood, Essex, UK), which aims at elevating the fetal head before a second-stage cesarean to diminish the risk of hysterotomy extension [28]. A recent meta-analysis was not able to conclude that any of these new techniques was superior to the standard techniques [25]. It is nonetheless possible that in centers with different obstetric practices, especially those with high rates of attempted operative vaginal deliveries before performing a cesarean, these methods might be useful. Other studies are necessary to reach a definitive conclusion.

## Conclusion

One in ten cesareans performed during the second stage of labor is complicated by a hysterotomy extension, itself a source of maternal complications. In our hospital, where delayed pushing is practiced, the risk of hysterotomy extension does not appear to be associated with any obstetric characteristics of the second stage of labor. Prolonging its passive phase beyond 2 or even 3 hours does not appear to increase the risk of hysterotomy extension when a cesarean delivery is finally performed.
